# Addressing physical skills and mental health: the role of modern teaching approaches in non-athlete university PE programs

**DOI:** 10.3389/fpsyg.2025.1664027

**Published:** 2025-12-05

**Authors:** Sumaira Aslam, Yongbin Shi, Chen Zhao

**Affiliations:** School of Physical Education, Henan University, Kaifeng, China

**Keywords:** non-athletes, physical education, mental health, skill development, teaching strategies, chronic diseases

## Abstract

**Background:**

Non-athlete university students often face limitations in developing physical skills due to traditional physical education (PE) teaching methods. Additionally, mental health issues like depression and anxiety are common among this group, and physical inactivity may worsen these problems, leading to chronic conditions such as cardiovascular diseases and diabetes. This research seeks to assess the effects of various PE teaching strategies on physical skill development and mental health outcomes, including depression and anxiety, among non-athlete University students in Henan Province.

**Methods:**

A hybrid method approach was employed, involving a stratified random sample of 220 students (116 male, 104 female, mean age, 20) and 16 physical education professors. Quantitative data were collected using a validated 35-item questionnaire (Cronbach’s *α* = 0.971) and structured observational checklists. Qualitative data were gathered through semi-structured interviews. Data was analyzed using SPSS 29.0 and NVivo 12. Student-centered teaching strategies, including cooperative learning (*β* = 3.802, *p* = 0.002) and peer teaching (*β* = 3.838, *p* = 0.002).

**Results:**

The study found a 22% improvement in physical skill competence and reductions in mental health issues, such as anxiety and depression. In contrast, traditional direct instructions yielded minimal impact. Institutional challenges, including large class sizes and obsolete facilities, hindered optimal implementation.

**Conclusion:**

This study highlights the efficacy of Student-centered teaching strategies in both enhancing physical skills and mental health outcomes among non-athlete students. It underscores the need to address institutional limitations for more effective (PE) programs and chronic disease prevention.

## 1 Introduction

Non-athlete university students face significant challenges, not only in physical skill development but also in mental health, often exacerbated by physical inactivity (Biddle et al., 2019; Rebar et al., 2015). Physical education (PE) interventions can play a pivotal role in addressing these challenges, enhancing both physical and mental well-being (Mahindru et al., 2023; Blankenship, 2017). These interventions have the potential to prevent chronic conditions, including cardiovascular diseases and diabetes, while fostering resilience and better coping mechanisms for mental health issues like anxiety and depression (Kelmendi and Dedi, 2024; Aksović et al., 2021; Erhorn et al., 2020). Teacher training has evolved to prioritize practices that align with students’ developmental needs, including recognizing how physical education can positively affect mental health outcomes (Ferry and Romar, 2020; Xin, 2021). Research indicates that high-quality physical education programs lead to increased student engagement (Hastie and Siedentop, 2006; Janssen and LeBlanc, 2010), better mood regulation, and enhanced skill acquisition (Siedentop and Van der Mars, 2022; Magee, 2013). This study assesses how various teaching methods, especially student-centered approaches, impact both skill development and mental health outcomes in non-athlete students, with a broader focus on long-term health benefits.

In China, where physical education is mandatory in public schools, instructors play a vital role in creating supportive environments for students (Kajanus, 2016; Shi, 2023). These environments are advised to incorporate diverse teaching methods to encourage active participation, skill development, and overall student well-being (Jaakkola and Watt, 2011). Poor teaching methods can lead to disengagement, exacerbating mental health issues and heightening the risk of physical problems (Somerset and Hoare, 2018; Suomi et al., 2003). These instructional strategies, implemented by physical education instructors, whether direct instruction, student-centered approaches, or collaborative learning, play a crucial role in enhancing students’ overall health and academic performance (Klapp et al., 2024; Felder and Brent, 2024).

Modern collaborative (student-centered) teaching methods have consistently been shown to be more effective than traditional direct instruction in promoting a wide range of student outcomes (Sangco, 2017). Evidence from educational research indicates that students taught through cooperative learning (CL) achieve greater motor skill gains and improved social–emotional outcomes compared to students taught by teacher-driven direct methods (Gumbo et al., 2017). Collaborative learning in PE classes actively engages students by building peer support networks and a sense of autonomy, which bolsters mental well-being. Additionally, recent studies confirm that structured peer group activities reduce student stress and emotional problems, ultimately providing measurable mental health benefits for learners (Capel and Whitehead, 2015; Shute, 2008; Van Ryzin and Roseth, 2021). This is particularly relevant for non-athlete students who often lack confidence or additional skills. A supportive, team-based learning environment helps alleviate anxiety and improves their attitude toward physical activity (de Carvalho and de Souza Neto, 2019; Casey and Goodyear, 2015; Cheng et al., 2019; Chu et al., 2021). In contrast, Direct Instruction (DI) is the traditional, teacher-centered approach, which excels in delivering well-organized drills and reinforcing the fundamental skills through repetition. DI has been associated with short-term improvements in specific motor skill performance; however, its one-way communicative nature may fail to address psychological engagement, which could lead to reduced motivation or heightened boredom, especially among older students. Rigid, instructor-led sessions may limit opportunities for peer interaction and personal agency, both of which are essential for supporting mental health and fostering student engagement. Critics note that while DI ensures structure, it does not inherently foster social support or enjoyment, which guards against anxiety among students. This contrast is particularly relevant in the Chinese university context, where direct instruction continues to dominate PE programs, and many non-athlete students perceive PE less as a supportive community activity and more as a compulsory routine (Jiang et al., 2023). By examining collaborative approaches alongside direct instruction in Chinese universities, this study addresses a critical gap in the literature. It is grounded in the premise that student-centered pedagogies can foster not only physical skill development but also psychological well-being, thereby contributing to the mitigation of depression and anxiety among college students (Han, 2021; Killen and O'Toole, 2023). The focus of this literature review positions our research within the ongoing empirical debate, with the expectation that modern, interactive teaching strategies will generate superior holistic outcomes compared to conventional methods, particularly for students who are not athletically inclined (Rezaei, 2020; Templin et al., 2016).

The effectiveness of physical education teaching practices enhances physical abilities and fosters positive attitudes toward exercise, thereby promoting lasting healthy habits and mental resilience. This research, therefore, examines the effect of various teaching techniques on enhancing physical skills and mental health outcomes, demonstrating the comprehensive benefits of well-structured physical education programs for university students.

### 1.1 Research problem

The significance of physical education (PE) in student development is recognized; however, the effectiveness of various teaching methods on improving skill competence and academic performance for non-athlete students remains unclear. Despite the various pedagogical approaches employed by physical education teachers, there is a lack of empirical data regarding the impact of these strategies on student engagement, skill acquisition, and overall academic success. Student attitudes, institutional resources, and teachers’ educational philosophies are likely factors influencing the effectiveness of these methods.

Furthermore, the mental health of non-athlete students, particularly symptoms of sadness and anxiety, significantly affects their motivation and participation in physical education. However, research is sparse on exploring how physical education teaching practices can address physical competence as well as mental health issues. This study aims to fill this gap by examining the relationship between teaching methods and both physical and psychological outcomes in non-athlete university students.

### 1.2 Objectives

To compare the effects of student-centered collaborative instruction compared to traditional direct instruction on the physical skill competence of non-athlete university students in PE classes. This objective focuses on measurable skill outcomes such as performance scores in standardized physical skill tests or assessments.

To evaluate the impact of collaborative. Direct teaching methods on students’ mental health, particularly levels of anxiety and depression, among non-athlete university students. This includes assessing changes in validated mental health inventory scores associated with various teaching approaches.

The study seeks to explore how moderating factors, namely instructor experience and resource availability, affect the efficacy of teaching methods. It specifically examines whether collaborative teaching is particularly beneficial in well-resourced environments and whether direct instruction performs relatively better when resources are limited.

This study aims to explore instructors’ and students’ perspectives on these teaching approaches through qualitative interviews and observations, thus contextualizing the findings. This approach illuminates practical challenges and institutional factors that influence the implementation of modern pedagogies.

### 1.3 Hypothesis

H1: University students using collaborative, student-centered methods will exhibit greater improvements in physical skill competence compared to using traditional direct instruction. This hypothesis predicts superior skill outcomes for the collaborative instructional group.

H2: Students in collaborative teaching PE classes will report better mental health outcomes, specifically, lower anxiety and depression scores on standardized scales, compared to direct instruction classes. Modern student-centered pedagogy is therefore expected to more effectively alleviate symptoms of anxiety and depression among non-athlete students.

H3: Institutional support and instructor profile will moderate the above effects. Collaborative teaching will yield the largest gains in student and mental health under well-resourced environments, compared to smaller gains in resource-limited settings. Additionally, younger instructors using collaborative techniques in supportive settings may achieve particularly strong improvements, while experienced instructors using structured direct methods. This study will examine whether the effectiveness of different teaching approaches varies between resourced and under-resourced settings.

### 1.4 Teaching strategies in physical education

#### 1.4.1 Cooperative learning

Cooperative learning (CL) is an educational approach prioritizing cooperation and peer engagement to enhance cognitive and social learning. One study has demonstrated that CL enhances academic achievement, social skills, critical thinking, and problem-solving abilities among students (Casey, 2016). Research indicates that students engaged in physical education who participate in cooperative exercises enhance their abilities, increase involvement, and improve social cohesiveness (Dyson, 2001; Metzler, 2017).

The effectiveness of cooperative learning is dependent on various factors, including student motivation and the quality of group dynamics. Research demonstrates that while CL often fosters peer connections, students demonstrating low motivation or poor interpersonal skills may find it challenging to participate, hindering group performance (Vasconcellos et al., 2020). Furthermore, the teacher’s involvement in group interactions is crucial. Unorganized groups and unclear instructions reduce the effectiveness of CL; organization and instructor facilitation are essential for CL success (Metzler, 2017).

In addition to enhancing physical abilities, cooperative learning significantly contributes to mental health and well-being. Fostering social connectivity and peer support, CL could reduce feelings of isolation and alleviate symptoms of depression and anxiety. The collaborative nature of CL creates a supportive atmosphere that enhances physical ability while encouraging psychological resilience and mental health outcomes (Kocak, 2008).

#### 1.4.2 Feedback in PE instruction

Feedback is a crucial component of effective physical education (PE) training, which enables students to assess and reflect on their performance. The concept of visual learning emphasizes the importance of making the process transparent for students and educators, which enhances understanding of student progress. Studies on feedback in physical education have demonstrated its crucial role in improving motor skills and conceptual understanding (Hattie and Timperley, 2007). Research indicates that students who receive specific, actionable feedback tend to develop their skills better than those who receive either minimal or no feedback (Kluger and DeNisi, 1996).

Furthermore, feedback that highlights effort and progress, rather than results, has been shown to foster a growing mindset, which enhances student engagement and tenacity (Felder and Brent, 2024). This approach fosters progress and proficiency, motivating students to persevere despite challenges. The frequency and type of feedback remain a contentious issue. Several studies support the findings that regular, formative feedback assists in facilitating student development (Shute, 2008; Han, 2021). Additional studies indicate that excessive input may result in cognitive overload, dissatisfaction, and diminished student performance (Xin, 2021; Hattie and Timperley, 2007).

Effective feedback requires structured implementation, including strategic timing and personalization, thereby maximizing its impact on student learning outcomes. Additionally, by fostering motor skill development, positive feedback promotes psychological well-being, self-efficacy increases their confidence, and reduces performance-related anxiety. This is particularly important for non-athletes, who may experience diminished self-esteem and heightened stress, as personalized feedback can improve their self-confidence and mental resilience.

#### 1.4.3 Direct learning approach

Direct Instruction (DI) is an approach characterized by well-organized classes and explicit teaching methods, which have been extensively researched in the context of physical education (PE). Study confirm its effectiveness in enhancing motor skill development and information retention (Halaidiuk et al., 2018). Research indicates that students who participate in DI surpass their peers in motor skill assessments, as this method emphasizes concentration and repetition, skill acquisition (Eppley and Dudley-Marling, 2019). Additionally, DI is assumed to enhance knowledge retention by offering systematic material delivery, enabling students to absorb and retain information over time.

Experts of DI argue that its rigid framework may restrain creativity and autonomy, potentially leading to disengagement, especially among older and advanced learners (Eppley and Dudley-Marling, 2019). While Direct Instruction (DI) may offer short-term effectiveness, it does not inherently promote problem-solving or independent thought, both of which are essential for developing critical thinking skills and adaptive learning (Mason and Otero, 2021; Stockard, 2015). Despite these limitations, recent research suggests that combining direct instruction with student-centered approaches, such as inquiry-based or cooperative learning, can improve educational outcomes (Pereira et al., 2016). This hybrid model employs DI’s systematic approach to build foundational knowledge while incorporating dynamic, interactive methods to enhance critical thinking and engagement (Wang et al., 2023; Ghavidel and Hamboushi, 2024).

Although research provides significant insights, several gaps remain. A significant portion of the research focuses on specific pedagogical strategies, such as feedback and cooperative learning, while overlooking their interaction in promoting holistic learning and growth. Additionally, research on CL often ignores the importance of student talent, motivation, and quality of cooperation, all of which are vital for the effectiveness of group activities (Pulgar et al., 2019). Furthermore, much of the research overlooks key contextual factors, such as student demographics, teacher proficiency, and the educational environment, all of which significantly impact instructional efficacy and the implementation of physical education programs.

Henan Province presents a unique environment for studying teaching methods in physical education, as the universities in this region are competitive, yet face significant financial constraints. Many public universities in Henan, like their counterparts across China, face challenges with inadequate facilities and teaching difficulties while striving to deliver high-quality education. Analyzing how PE instructors in Henan address these issues offers valuable insights into effective teaching strategies in under-resourced settings and may inform practices in similar contexts elsewhere.

### 1.5 Research gap

This study focuses on the impact of instructional methods on skill development and mental health outcomes in non-athlete learners within physical education (PE) is significantly limited. This gap highlights the need for additional research on various instructional methods on students’ proficiency, engagement, and psychological well-being. Research demonstrates that PE instruction not only enhances skill acquisition but additionally supports students’ mental health by alleviating symptoms of sadness, anxiety, and stress, while fostering an attitude toward physical exercise (Kirk, 2009). Effective pedagogical methods may increase engagement and enjoyment in physical education, thereby promoting both physical and mental well-being (Bailey, 2006). Nonetheless, identifying strategies that address these diverse needs of non-athlete students, particularly those facing mental health challenges, remains a significant challenge.

Henan province non-athlete university students often encounter mental health issues, such as apathy, unfavorable perceptions of physical exercise, diminished self-esteem, and relatively poor physical fitness, often with intensified stress, anxiety, and depressive symptoms (Casey and Goodyear, 2015; Trudeau and Shephard, 2008). These lead to disengagement and the formation of unfavorable attitudes toward physical exercise, thereby increasing the risk of mental health disorders and physical ailments owing to inactivity. The conventional physical education curriculum, centered on competitive sports and standardized skill evaluations, may be insufficient in addressing the mental health requirements of these students, underscoring the need for pedagogical approaches that foster psychological well-being, inclusivity, and active participation in physical education environments.

This research aims to address this gap by evaluating the effectiveness of various teaching methods in improving physical education programs for non-athlete students. The goal is to enhance their physical abilities and mental health outcomes, simultaneously reducing stress, anxiety, and the risk of depression associated with sedentary lifestyles.

### 1.6 Contextual factors

The need to investigate the correlation between pedagogical approaches and student achievement and engagement in physical education (PE) is increasing. Motivation plays a crucial role in influencing students’ readiness to participate in physical exercise and enhancing their abilities (Haerens et al., 2015). In addition to motivation, other critical factors affecting the effectiveness of teaching methodologies in physical education include class size, resource availability, teacher credentials, and the school environment (Kelmendi and Dedi, 2024; Gumbo et al., 2017). Additionally, an educator’s age, experience, and ongoing professional development are also essential considerations. Younger, well-trained, and educated educators tend to be more skilled at employing diverse and effective pedagogical strategies. The environment, including administrative support and the prioritization of physical education within the broader curriculum, has a significant impact on the quality of physical education instruction (Killen and O'Toole, 2023; Lipponen et al., 2022; Souza e Silva et al., 2023).

Mental health issues, including depression, anxiety, and stress, are significant contextual variables that may influence students’ willingness to engage in physical education. Students facing these mental health challenges often display a loss of energy, decreased and detachment in physical exercise, which hinders skill development (Stevens et al., 2020). These mental health obstacles negatively impact their physical participation and overall academic performance in physical education. Therefore, addressing mental health through supportive teaching practices, emphasizing inclusion, and creating a positive environment is essential for fostering engagement and skill development. Integrating mental health support into physical education enhances student engagement, mental well-being, leading to better physical and psychological outcomes.

## 2 Methodology

### 2.1 Study design

This research employed a mixed-method study to explore the influence of PE instructors’ teaching tactics on skill development, motivation, and mental health outcomes among non-athlete students’ universities in the province of Henan. This method facilitates data triangulation, thereby enhancing the validity of the results (Creswell and Creswell, 2017). The quantitative component comprises a self-designed questionnaire that evaluates the impact of instructional approaches on physical abilities, engagement, and mental health. The qualitative component includes semi-structured interviews with physical education instructors to gain insight into their perspectives on the effect of instructional practices on physical and mental well-being. Additionally, observational data were collected during physical education sessions to document real-time instructional tactics and their effects on students’ physical and psychological outcomes.

This approach provides a comprehensive understanding of the impact of instructional tactics on the physical competence and mental health of non-athlete students, offering valuable insights for enhancing physical education programs.

### 2.2 Study subjects and sample selection

We employed a stratified random sampling strategy to recruit student participants, ensuring that key subgroups were represented. Strata were defined by the academic discipline (science majors vs. Liberal Arts majors) and by academic year (first-year vs. second-year students), creating four strata in total: (1) Science-Year 1, (2) Science-Year 2, (3) Arts-Year 1, and (4) Arts-Year 2. Only ‘non-athlete’ students, defined as those not majoring in sports/physical education and not members of the university sports team’s enrollment lists, with approximately equal numbers per stratum (about 50–60 students), resulting in *N* = 220 students (57% first-year, 43% second-year). This approach ensured proportional representation across faculties and year levels. In addition, 16 Physical education instructors from the universities were initially approached, with 10 consenting to participate in the qualitative components (Guthold et al., 2018). These represented a range of ages, ranks, and sports specializations. Table 1 illustrates the distribution, collection, and effective collection rates of questionnaires across various institutions in the province of Henan. The figures indicate the effectiveness of data collection, revealing a high collection rate (100% for most institutions) of valid responses. The effective collection rate, which indicates the proportion of legitimate questions, serves as a crucial evaluation of the survey’s reliability and validity.

**Table 1:** Demographics of interview participants at Henan University.

Participant’s					
ID	Age	Gender	Years of experience	Job title	Sports
P1	60	Male	20	Professor	Fitness
P2	55	Female	24	Assis Prof	Aerobic ex
P3	30	Female	3	Lecturer	Aerobic ex
P4	56	Male	16	Assis Prof	Badminton
P5	55	Male	20	Assis Prof	Badminton
P6	37	Male	7	Lecturer	Fitness
P7	60	Male	25	Lecturer	Football
P8	58	Male	20	Assis Prof	Football
P9	40	Male	10	Assis Prof	Tennis
P10	50	Male	17	Assis Prof	Basketball

Priori power analysis (G*Power 3.1) yielded a sample size of 220 students, which provided sufficient statistical power (0.9995) to detect a medium effect size (*f*^2^ = 0.15) across all quantitative analyses, including correlations, regressions, and chi-square tests, at *α* = 0.05. This ensured robustness in identifying significant relationships and effects within the research.

Instructors were intentionally selected based on faculty position (professors, associate professors, lecturers), teaching experience (3–25 years), and sports specialization (e.g., team sports, fitness, and individual sports) to encompass a range of instructional methodologies and institutional challenges. This ensured representation of traditional and progressive pedagogical approaches, aligning with the study’s focus on contextual moderators of strategy effectiveness. Informed consent in writing was obtained prior to data collection.

Qualitative data collection: Semi-structured interviews were conducted with 16 purposively selected PE instructors from Henan province universities, representing diverse sports specializations, academic ranks, and 3–25 years of experience, to explore teaching philosophies, strategy use, and contextual challenges. The interview guide, developed from literature and expert input (Delphi panel, *n* = 12), detailed in Appendix C, included demographics, pedagogical approaches, professional development, technology use, and implementation barriers. To ensure rigor, a standardized protocol, interviewer training, and consistent sequencing were employed. Construct and face validity were confirmed through expert review, and findings were triangulated with questionnaire and observational data. Interviews, lasting 30–45 min, were conducted in private settings, audio-recorded with consent, transcribed verbatim, and thematically analyzed in NVivo 12 following Braun and Clarke’s six-step framework, with inter-rater reliability checks to enhance coding consistency.

Rationale for ordinal regression: Ordinal regression was employed because the dependent variables, such as mental health scores and physical skill competence, were measured on an ordinal scale (ranging from ‘very low’ to ‘very high). Ordinal regression is appropriate for analyzing ordinal categorical outcomes while accounting for the relationships between predictors and outcome variables. The model enables us to predict the likelihood of various outcomes based on teaching strategies, student engagement, and other demographic factors.

Handling missing data: Missing data were handled using multiple imputation in SPSS 29.0. This method is appropriate for maintaining statistical power and minimizing biases caused by missing values. Imputation was performed based on available data, and sensitivity analyses were conducted to verify that the imputation process did not introduce significant bias into the results.

Reporting effect sizes: In addition to *p*-values, effect sizes (Cohen’s *f*^2^ for regression analysis) were reported to assess the practical significance of the findings. This is important because *p*-values alone do not provide information about the magnitude of an effect, which is crucial for understanding the practical importance of the teaching methods. For example, the *β* coefficient for cooperative learning (*β* = 3.802, *p* = 0.002) was accompanied by Cohen’s *f*^2^ = 0.27, indicating a medium effect size. Similarly, peer teaching (*β* = 3.838, *p* = 0.002) had an effect size of Cohen’s *f*^2^ = 0.24, indicating a moderate effect. Reporting effect sizes in addition to *p*-values provides a more comprehensive understanding of the study’s results and their practical significance.

#### 2.2.1 Demographic characteristics of participants

The demographic breakdown (Table 2) indicates that male participants (68%) compared to females (31%), had a higher proportion of teachers and students. Age distribution was predominantly 20 years old (79.5%), while all teachers were aged 30–60 years, emphasizing a clear distinction between both groups. The teaching experience varied significantly, with half of the instructors having over 20 years of experience, whereas a small fraction (10%) had less than 5 years. Among students, first-year participants (57%) were slightly more represented than second-year students (42%) (Table 3).

**Table 2:** Demographic summary of study participants.

Characteristic	Teachers (*n* = 10)	Students (*n* = 220)	Total (*n* = 230)
Gender			
Gender (male)	8	116	124(68%)
Gender (female)	2	104	106(31%)
Age group			
Age group (21–25)	0	13	13
Age group (20)	0	175	175
Age group (18)	0	32	32
Age group (30–60)	10	0	10
Teaching experience			
Teaching experience (<5 years)	1	N/A	1
Teaching experience (5–10 years)	2	N/A	1
Teaching experience (11–19 years)	2	N/A	2
Teaching experience (>20 years)	5	N/A	5
Grade level (students)			
1st Year	N/A	127	127(57%)
2nd Year	N/A	93	93(42%)

**Table 3:** Distribution, acquisition, and efficacy of questionnaire collection rates over participant universities.

University	Questionnaires distributed	Questionnaires collected	Valid questionnaires	Invalid questionnaires	Collection rate (%)	Effective collection rate (%)
Henan University (Students)	220 (Students)	220	220	0	100%	100%
Henan University (Teachers)	16 (Teachers)	16	16	0	100%	100%
Zhengzhou University	60	60	58	2	100%	96.7%
Henan Normal University	60	60	56	4	100%	93.3%
Luoyang Normal University	60	60	58	2	100%	96.7%
Henan University of Science (2013)	16 (2013) + 18 (2012)	16 (2013) + 16 (2012)	16 (2013) + 14 (2012)	0 + 2	94.1%*	88.2%*
Henan University of Science (2012)	16 (2013) + 16 (2012)	16 (2013) + 16 (2012)	16 (2013) + 14 (2012)	0 + 2	94.1%*	88.2%*
Anyang Normal University	15	13	13	0	86.7%	86.7%
Shangqiu Normal University	20	20	16 + 17	4	100%	82.5%
Zhoukou Normal University	243	227	227	16	93.4%	93.4%

Table 1 indicates the distribution, collection, and effective collection rates of questionnaires across various colleges. It specifies the quantity of questionnaires disseminated, retrieved, and deemed legitimate for each institution, together with the corresponding collection rates. The data indicates a high success rate in collecting, with a few attributed to incorrect questionnaires. The effective collection rate column reflects the proportion of valid replies obtained, providing insights into the reliability of the survey findings across other colleges.

Table 1 indicates the demographic attributes of 10 faculty members from Henan University, including age, gender, years of experience, academic position, and sports specialization. The participants have 3- to 25 years of experience, with most holding senior positions. The age distribution indicates that most participants were 50 years or older, reflecting extensive professional experience. The sample is predominantly male (*n* = 8), with two female participants, specializing in aerobic exercise. Faculty members were involved in a range of PE classes, and this distribution highlights diverse expertise across individual and team sports, ensuring broad representation in physical education instruction.

### 2.3 Data collection methods

#### 2.3.1 Questionnaire development

The questionnaire used in the study was adapted from validated scales to ensure reliability and validity. Items related to student engagement and teaching strategies were drawn from a scale in educational research (Hattie and Timperley, 2007). Adaptation involves modifying the language to align with the specific context of PE. The development process for new items included consultations with subject matter experts through the Delphi method. A pilot study was implemented to test clarity, relevance, and reliability.

#### 2.3.2 Reliability and validity tests of questionnaire and interview items

The initial draft of the student questionnaire conducted a pilot test with 30 non-participant students from a similar population. Feedback from this pilot study was used to rectify all items. Furthermore, the survey instrument was validated through expert review using a Delphi method (Niederberger and Spranger, 2020; Nasa et al., 2021). Two rounds of evaluation by a panel of five experts in PE and sports psychology led to improvements in content validity. The final questionnaire comprised of 35 items. Reliability analysis on the study sample showed a Cronbach’s alpha of 0.971, indicating excellent internal consistency. We also assessed construct validity: the Kaiser–Meyer–Olkin (KMO) measure was 0.801 and Bartlett’s test of sphericity was significant (*p* < 0.001), supporting the ability of the survey data and the adequacy of our sample for factor analysis. These steps confirmed that the questionnaire was reliable and valid for measuring the targeted outcomes. No pilot was necessary for the interview guide, as those questions were reviewed by experts during the Delphi process and refined for clarity; however, interviewers conducted two trial interviews with colleagues to practice the protocol, which finalized the interview approach.

#### 2.3.3 Observational data collection

A structured rubric was used to analyze instructional tactics in the observational data, based on frameworks from prior research (Dyson, 2001; Metzler, 2017). The rubric included elements such as instructional clarity, student involvement, and feedback provision. Observers participated in training sessions to ensure consistency and accuracy in the application of the rubric. Inter-rater reliability was assessed using Cohen’s kappa, yielding a score of 0.85, indicating a substantial degree of agreement. Observational results were corroborated by questionnaire and interview data to enhance the reliability and depth of the research. These findings clarified the impact of teaching tactics on physical skill development and their role in mitigating mental health challenges, including stress and anxiety, by fostering a positive learning environment.

### 2.4 Ethical considerations

All the study protocols were approved by the university’s ethical review and research board [Henan University Institutional Review Board (IRB)]. Informed consent was given by all participants. Participants were informed about the risks and benefits of participation in the study. All participants were anonymized to protect their identities. Personal information, such as age and gender, was collected only to contextualize responses, and all data which were stored securely. Participation in the study was entirely voluntary. Participants were also informed that they could withdraw from the study at any point without any consequences.

### 2.5 Data analysis approach

Qualitative data were analyzed using descriptive statistics (means, standard deviations), and appropriate inferential statistics were aligned with our research questions. We computed Pearson correlation coefficients to examine associations between instructional tactics and student outcomes, and performed Pearson chi-square tests to assess categorical differences in teaching strategy usage across observations. Furthermore, we conducted ordinal logistic regression analyses to identify which teaching strategies and sports significantly predicted student effectiveness ratings. All quantitative analyses were conducted in SPSS 29.0 with a significant level of *α* = 0.05. For each inferential test, we report the test statistic, *p*-value, and an effect size (Cramer’s *V* for chi-square tests; regression coefficient *β* for ordinal regression) along with 95% confidence intervals to convey the precision and practical significance of the results.

Qualitative interviews and observational data were analyzed thematically using the six-step framework by Braun and Clarke (2006) and Braun and Clarke (2019) with NVivo 12 software. This procedure included data familiarization, code generation, topic identification, review, definition, and the production of the final report. Thematic coding focused on patterns related to instructional adaptation, student engagement, and the effectiveness of feedback. The results from both data sets were integrated to provide a comprehensive understanding of the impact of instructional strategies on physical competence and mental health, specifically in reducing anxiety and stress.

## 3 Results

### 3.1 Quantitative analysis

#### 3.1.1 Descriptive statistics

The descriptive statistics summarize students’ perspectives on the efficacy of diverse instructional styles on enhancing their skill development and mental health outcomes. Students were asked broad questions about their understanding of pedagogical strategies, effective learning, and their overall view of PE. The items were rated using a Likert scale from 1 (extremely ineffective) to 5 (highly effective). Questions regarding challenges in physical education courses and the impact of instructional styles on learning outcomes were included. The questionnaire consisted of three sections: demographic characteristics, pedagogical methodologies, and student learning experiences.

Table 4 indicates that clarity of teaching tactics received a favorable mean score of 4.05, suggesting that students mostly understood the strategies. The significantly high standard deviation (0.910) reflects variability in responses. Conversely, students’ assessment of the effectiveness of drills and exercises (mean = 2.74) indicates a need for improvement in communicating the objectives of these activities. The mean score of 1.90 for tactics aimed at enhancing abilities and performance suggests that students perceive these approaches as ineffective, emphasizing the need to develop these strategies for improved student engagement and learning outcomes. The view of individual feedback (mean = 4.21) emphasizes that students value feedback and its potential impact on their self-confidence and motivation, which are crucial for reducing anxiety and stress. Additionally, the use of technology (mean = 4.17) was regarded as beneficial, possibly alleviating mental health issues by providing students with tools to visualize their performance and receive prompt feedback, thereby decreasing performance anxiety.

**Table 4:** Effect of PE teaching strategies on physical skill competence and mental health outcomes.

Strategies	Mean SD	Median	Mode	Std. deviation
Clarity of the PE teaching strategies	4.05 (0.910)	4.00	5	0.910
Specific drill or exercise	2.74(0.711)	3.00	3	0.711
Strategies Improving skills/performance	1.90(0.666)	2.00	2	0.666
Performance feedback	2.33(1.013)	2.00	2	1.013
Effective teaching strategies	2.02(0.960)	2.00	1	0.960
Individual feedback	4.21(0.908)	4.00	5	0.908
Use of technology tools.	4.17(0.900)	4.00	5	0.900
Enough opportunities to ask questions	1.26(0.839)	1.00	1	0.839
Challenging activities	1.97(0.381)	2.00	2	0.381

This table summarizes the impact of different teaching strategies on physical skill development and mental health outcomes in non-athlete students.

Students reported limited opportunities for inquiry (mean = 1.26) and viewed the activities as less challenging (mean = 1.97). The lack of engagement may lead to mental health issues, such as apathy and stress, due to feelings of neglect or unstimulating activities to maintain their involvement.

Table 5 indicates teacher communication strategies (mean = 1.66) and the comfort level of asking for clarification (mean = 1.99) received low ratings. These findings suggest that students may feel mentally disengaged or anxious due to insufficient communication or opportunities to seek help. The lack of clarity and support could increase stress and anxiety, as students might feel uncertain about their understanding of the material. Similarly, the feedback provided was not perceived as very helpful (mean = 1.73), indicating a need for more targeted and supportive feedback to alleviate feelings of confusion and frustration, which often lead to anxiety and stress.

**Table 5:** Descriptive statistics of mean measure.

Teaching strategy	Mean (SD)	Median	Mode	Std. deviation
Teachers communicate strategies clearly	1.66 (0.738)	2.00	1	0.738
Comfortable asking for clarification	1.99 (0.802)	2.00	2	0.802
Feedback provided helps understand the PE teaching strategies better.	1.73 (0.713)	2.00	2	0.713
Teaching strategies make PE classes more interesting and enjoyable.	2.57 (1.465)	2.00	2	1.465
Diverse activities.	4.31 (0.879)	5.00	5	0.879
Equal opportunities to learn and participate effectively.	1.24 (0.426)	1.00	1	0.426

In contrast, diverse activities (mean = 4.31) were rated positively, indicating that varied teaching strategies may enhance engagement and provide students with a more stimulating learning environment. This could be beneficial in addressing mental health issues like depression and disengagement, as engaging in activities may foster a sense of accomplishment and alleviate negative emotional states.

#### 3.1.2 Inferential statistics

Table 6 indicates a modest positive association (*r* = 0.323) between student learning (SL) and teaching techniques (TS), suggesting that improvements in teaching methods are associated with better student learning outcomes. This association is statistically significant (*p* = 0.000), indicating that effective teaching practices are favorably correlated with improved learning results and mental well-being. Teaching techniques that encourage engagement and skill development can reduce tension and anxiety by setting clear objectives and fostering a supportive learning environment.

**Table 6:** Correlation Coefficient of SL and TS.

Variables	SL	TS
SL	1	0.323**
TS	0.323**	1
Sig. (2-tailed)		0.000
*N*	220	220

Correlation is significant at the 0.01 level (2-tailed).

#### 3.1.3 Chi-square test

Tables 7, 8 indicates that cooperative learning and peer teaching were extensively used, while strategies such as skill breakdown and repetition of practice were less utilized. Cooperative learning and peer teaching significantly enhance mental health by promoting social engagement, alleviating anxiety, and bolstering self-confidence through the establishment of supportive peer settings.

**Table 7:** Frequency of teaching strategy usage in round 1.

Strategy usage	R1			
	Low	Moderate usage	High usage	Total
Cooperative learning	0	0	16	16
Feedback	4	2	9	15
Peer teaching	0	1	14	15
Repetition of practice	1	11	3	15
Skill Breakdown	13	1	1	15

**Table 8:** Frequency of teaching strategy usage in round 2.

Strategy	R2			
	Low usage	Moderate usage	High usage	Total
Cooperative learning	0	0	16	16
Feedback	5	6	5	16
Peer teaching	1	1	14	16
Problem-solving	4	2	5	11
Skill-Breakdown	9	2	1	12

Table 9 shows that the prevalence of Cooperative Learning remained at 100% (16/16), with peer teaching also showing improvement. These tactics enhance mental well-being by increasing engagement and fostering peer support, simultaneously reducing anxiety and sadness. Conversely, tactics such as Skill breakdown showed little utilization, which may limit opportunities for comprehensive skill learning and result in disengagement, thereby exacerbating mental health concerns.

**Table 9:** Chi-square test round 1 and 2.

Test	Round 1	Round 2
Pearson chi-square	358.225**	104.882**
Degrees of freedom	68	48
Asymptotic significance	0.000	0.000
Likelihood ratio	191.895	133.870
*N* of valid cases	170	165

Table 10 presents the chi-square test results from two rounds of observational data. The Pearson Chi-Square values for round 1 (358.225) and round 2 (104.882) indicate a stronger association between the categorical variables. The drop in Round 2 suggests that, while the relationship between teaching strategies and student outcomes remains significant, teaching strategies have a significant influence on student learning outcomes in both rounds (*p* = 0.000). The strength of this relationship was stronger in Round 1 but remained statistically significant in Round 2. The slight reduction in Chi-Square and Likelihood Ratio values in Round may suggest that some variables became less influential over time, or students became more accustomed to the teaching method.

**Table 10:** Ordinal regression analysis of R1 sports.

Parameter estimates of PE classes								
		Estimate	Std. error	Wald	df	Sig.	95% confidence interval	
							Lower bound	Upper bound
Sport	Aerobic exercise	0.034	0.991	0.001	1	0.973	−1.908	1.976
	Badminton	2.015	1.011	3.975	1	0.046	0.034	3.996
	Basket ball	0.152	1.033	0.022	1	0.883	−1.873	2.177
	Fitness	1.013	1.097	0.853	1	0.356	−1.137	3.163
	Foot ball	0.452	1.050	0.185	1	0.667	−1.605	2.509
	Tennis	0.301	0.993	0.092	1	0.762	−1.645	2.247
	Volley ball	0.117	1.032	0.013	1	0.910	−1.907	2.141
	Wushu	0^a^						

^a^ Descends the reference category in the ordinal regression model.

**Figure 1:**
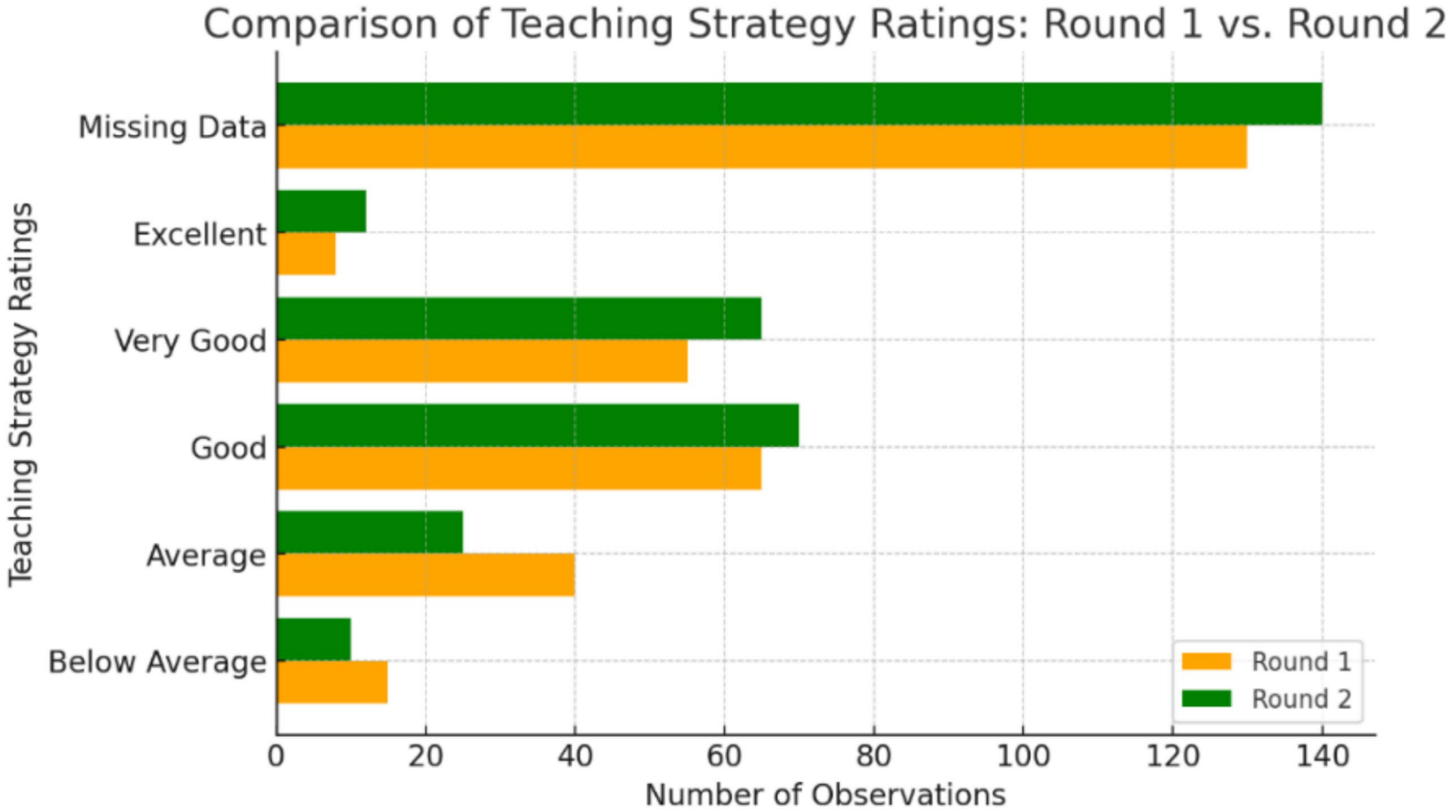
Comparison of outcomes between student-centered and direct instruction.

Figure 1 indicates evaluations from two rounds (R1 and R2), and assesses the level of strategies used in PE classes. The methods are categorized as (below average, good, very good, and excellent) based on their usage. If a strategy was not used, it was considered missing. Strategies with limited usage are rated as below average. The ratings were determined by the maximum usage observed in both rounds. The second round showed an increase in ‘Excellent’ and ‘Very Good’ ratings, and a decrease in ratings below average and average, indicating overall improvement. Good ratings remained consistent in both rounds. The figure suggests an improvement in overall ratings from Round 1 to Round 2.

#### 3.1.4 Regression analysis

Table 11 shows an ordinal regression analysis which was performed to determine the relationship between different sports and their impact on effectiveness during PE classes, with round 1 as the outcome variable. The results indicate that individual sports such as badminton and fitness had a significant positive impact on effectiveness in Round 1, with estimates of 1.874 and 1.059, respectively. These values have a statistical Significance of *p* = 0.050 and *p* = 0.062 Indicating their meaningfulness. However, other sports, such as team sports, football, basketball, tennis, and aerobic exercise, do not show significant associations, as their *p*-values are higher than 0.05. Table 11 shows a significant positive effect on effectiveness, indicating that individual sports, such as badminton, create an environment that reduces anxiety and enhances involvement, which may be associated with improved mental health outcomes.

**Table 11:** Estimated parameters of teaching strategies.

Parameter estimates of strategies								
Strategies		Estimate	Std. Error	Wald	df	Sig.	95% confidence Interval	
							Lower Bound	Upper Bound
	Challenge tasks	−2.556	1.645	2.415	1	0.120	−5.780	0.668
	Cooperative learning	3.802	1.226	9.621	1	0.002	1.400	6.205
	Demo	1.876	1.174	2.553	1	0.110	−0.425	4.178
	Feedback	1.683	1.190	1.998	1	0.158	−0.651	4.016
	Goal oriented	−1.204	1.323	0.828	1	0.363	−3.797	1.390
	Individual instruction	−0.026	1.184	0.000	1	0.982	−2.347	2.294
	Inducing new techniques	−35.064	2145.039	0.000	1	0.987	−4239.263	4169.134
	Peer teaching	3.838	1.228	9.775	1	0.002	1.432	6.244
	Problem solving	2.171	1.211	3.214	1	0.073	−0.203	4.545
	Reflective practice	−0.111	1.352	0.007	1	0.934	−2.761	2.538
	Repetition of practice	0.755	1.171	0.416	1	0.519	−1.539	3.050
	Scaffolding instruction	−0.777	1.420	0.299	1	0.584	−3.560	2.007
	Skill breakdown	−2.980	1.307	5.203	1	0.023	−5.541	−0.419
	Station teaching	0.808	1.183	0.467	1	0.494	−1.510	3.126
	Targeted dills	−0.882	1.838	0.230	1	0.631	−4.484	2.720
	Training modifying strategies	−0.724	1.607	0.203	1	0.652	−3.874	2.426
	Use of technology	−0.542	1.812	0.089	1	0.765	−4.092	3.009
	When and how to use a technique	0^a^			0			

^a^ Descends the reference category in the ordinal regression model.

Table 12 indicates an ordinal regression analysis, which analyzed the effectiveness of various pedagogical practices seen in physical education classrooms. The coefficient for ‘inducing new technique’ (−35.064) is unusually large, suggesting potential issues with model stability or multicollinearity. This result requires further scrutiny, as it may reflect data anomalies or inappropriate model specifications. Given that the study involves multiple tests, there is an increased risk of Type I error. To mitigate this, we applied the Bonferroni correction for multiple comparisons. However, the extreme coefficient suggests that further model validation and sensitivity analyses are necessary to confirm the robustness of these results. Additionally, future research using longitudinal or experimental designs could help clarify the causal relationships between teaching methods and student outcomes.

**Table 12:** Estimated parameters of PE sports.

Parameter estimates of PE sports								
Sport		Estimate	Std. error	Wald	df	Sig.	95% confidence interval	
							Lower bound	Upper bound
	Aerobic exercise	0.034	0.991	0.001	1	0.973	−1.908	1.976
	Badminton	2.015	1.011	3.975	1	0.046	0.034	3.996
	Basket ball	0.152	1.033	0.022	1	0.883	−1.873	2.177
	Fitness	1.013	1.097	0.853	1	0.356	−1.137	3.163
	Foot ball	0.452	1.050	0.185	1	0.667	−1.605	2.509
	Tennis	0.301	0.993	0.092	1	0.762	−1.645	2.247
	Volley ball	0.117	1.032	0.013	1	0.910	−1.907	2.141
	Wushu	0^a^			0			

^a^ Descends the reference category in the ordinal regression model.

In Table 13, a regression analysis was performed to explore the connection between various sports and teaching strategies and their impact on effectiveness in Round 2. Similar to Round 1, badminton and fitness demonstrated a significant positive correlation with Round 2, with parameter estimates of 2.015 and 1.013, respectively, and *p*-values of 0.046. This suggests that badminton and fitness are positively linked to increased effectiveness in Round 2. On the other hand, team sports like basketball, football, tennis, and volleyball in physical education classes did not yield significant p-values, indicating no difference in teaching strategies for Round 2.

**Table 13:** Significant predictors of effectiveness in round 2.

Strategy	*β*(SE)	Wald	*p*-value	95% confidence interval
Challenge tasks	−3.31 (1.41)	5.52	**0.019**	**[−6.07, −0.55]**
Cooperative learning	2.03 (1.24)	2.67	0.102	[−0.41, 4.47]
Peer teaching	**1.74 (1.23)**	**2.01**	**0.157**	**[−0.67, 4.15]**
Skill breakdown	−3.56 (1.30)	7.58	**0.006**	**[−6.10, −1.03]**

Values in bold italic indicate statistical significance at *p* < 0.05.

In Table 14, Skill breakdown (*β* = −3.56, *p* = 0.006) and challenge tasks (*β* = −3.31, *p* = 0.019) negatively impacted effectiveness in Round 2, indicating overuse or misapplication. Peer teaching showed a positive but non-significant trend (*β* = 1.74, *p* = 0.157). Cooperative learning’s non-significance (*p* = 0.102) contrasts with its high usage, suggesting contextual limitations.

**Table 14:** Themes output of interviews.

Themes	Files	References
(RQ1) teaching strategies	10	127
(theme1) Strategies employed by PE teachers	10	114
Teaching strategies and practice	10	114
Personal and professional development	10	81
(RQ2) significant correlation	10	112
(theme2) Teaching philosophy influences learning outcomes	10	96
Teaching Philosophy	10	96
Assessment	10	42
Technology integration	10	29
(RQ3) Factor of affecting performance outcomes	10	37
(Theme3) inadequate resources and challenges	10	34
Students’ diversity	10	18
Workload and resources	9	16

### 3.2 Qualitative analysis

Interviews were conducted with 10/16 experienced PE teachers with varying years of experience and expertise in different sports (Table 14). The data was collected through semi-structured interviews, which allowed flexibility in exploring key themes while ensuring that all participants addressed similar topics. The interview included questions on the instructional strategies used in their physical education (PE) classes, Perceptions of students’ engagement and learning outcomes, Challenges faced in implementing specific strategies, Reflections on professional development, and new teaching methods. The interview data were analyzed using NVivo software, facilitating the identification of codes, themes, and patterns. These semi-structured interviews with PE teachers delved deeply into their teaching strategies, student engagement, resource constraints, and instructional philosophy.

#### 3.2.1 Thematic analysis

In Table 14, the data revealed that 127 references related to teaching strategies specifically emphasized interactive methods such as cooperative learning, peer teaching, and personalized feedback. Cooperative learning was consistently recognized as one of the most effective ways to enhance student engagement, as indicated by 16 interviews. Educators noted that cooperative learning promotes collaboration, allowing students to learn from one another while strengthening social interaction and teamwork. A participant indicates that CL fosters student confidence as they collaborate to attain shared objectives in fitness activities. Peer teaching was another frequently used approach. Educators have discovered that students often respond positively to their peers, which boosts motivation and engagement. One participant indicates that when students instruct one another, comprehension improves, and they take greater responsibility for their learning. Furthermore, educators emphasized the importance of providing individualized feedback during physical activities. Tailored feedback was considered essential for helping students refine their techniques. Another participant observed that offering feedback during exercises helps students correct their form and boosts their confidence in their abilities. However, challenges were noted in delivering personalized feedback, particularly in larger classes. A participant said, ‘Prompt corrections are essential, yet large classes hinder personalization. This issue led to a significant decline in the effectiveness of feedback due to resource limitations. These findings emphasize the importance of CL methodologies and individualized feedback in enhancing student engagement and performance.

In contrast, Table 14 interviews highlighted the positive impact of cooperative learning and peer instruction on student engagement. Educators observed that these tactics significantly enhanced students’ self-esteem, alleviated stress, and fostered a sense of community, which is beneficial for mental health. Personalized feedback was seen as essential for enhancing both physical abilities and emotional well-being, particularly in helping students alleviate performance anxiety.

**Figure 2:**
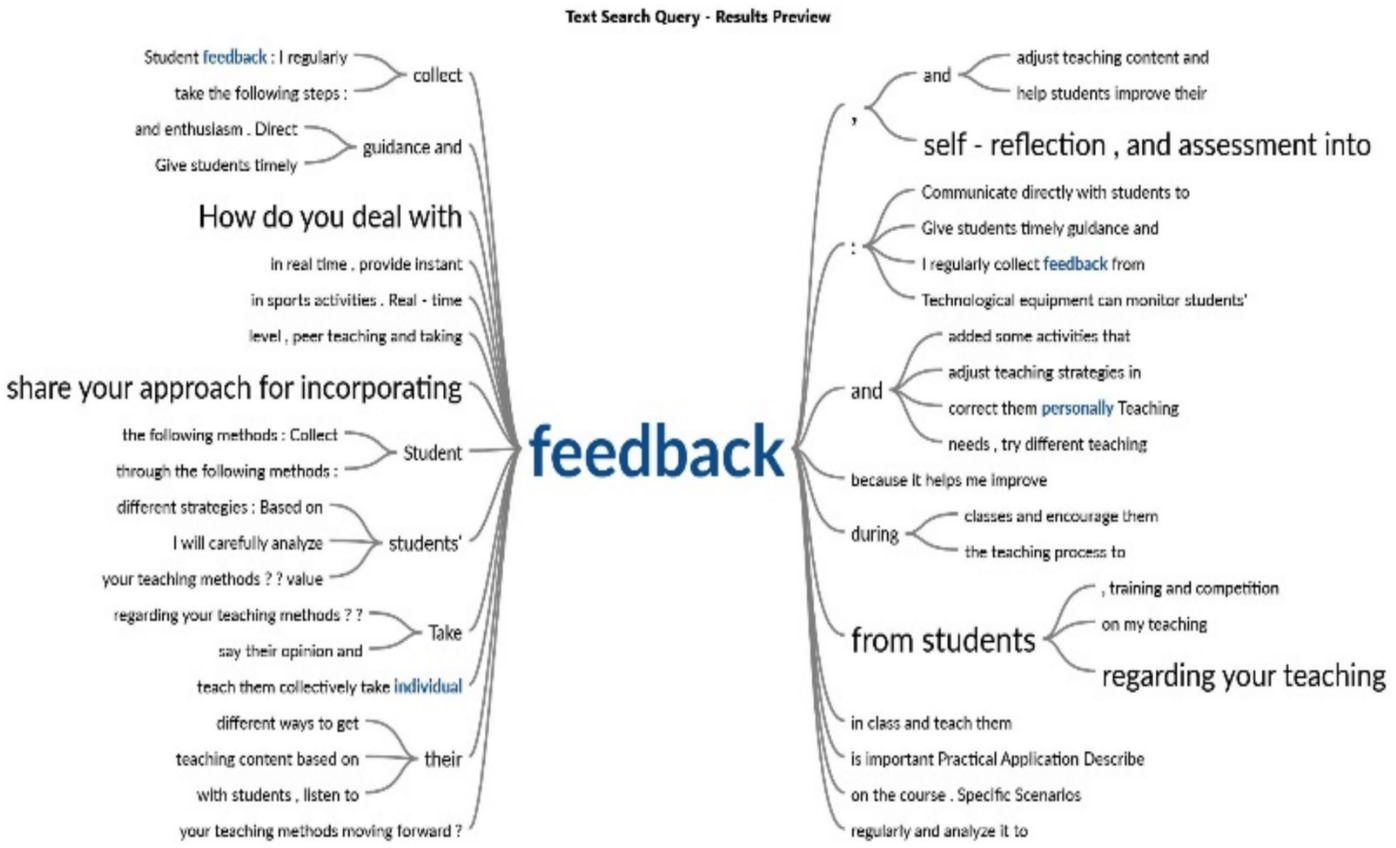
Personalized feedback.

As shown in Figure 2, the process of collecting and integrating feedback is essential for refining teaching methods. Regularly incorporating student feedback through various channels, such as peer teaching and direct responses, enables instructors to adjust their teaching strategies and improve learning outcomes.

#### 3.2.2 Teaching philosophy and its influence on learning outcomes

A substantial association exists between educators’ ideologies and student results, with 96 references demonstrating that student-centered and active learning methodologies improve engagement, performance, and mental well-being. Educators who prioritized student needs showed increased enthusiasm and engagement, fostering a conducive learning atmosphere. A participant mentioned that such as, “Emphasizing students’ needs and fostering active learning substantially enhances participation,” which helps mitigate performance anxiety. The use of technology, video analysis, and fitness applications further increased engagement and alleviated stress by enabling students to observe their progress. These results highlighted the efficacy of student-centered pedagogy and technological integration in enhancing academic performance and psychological well-being.

**Figure 3:**
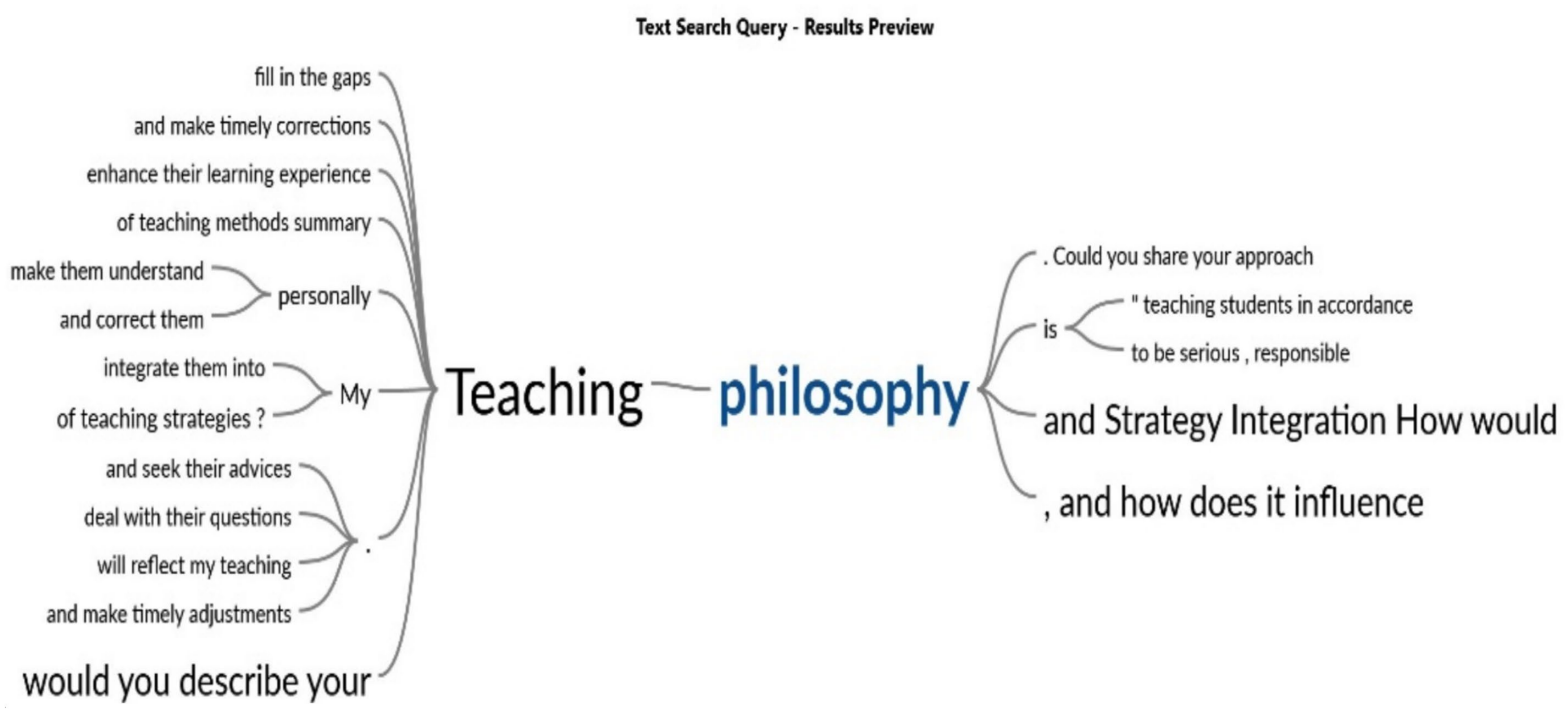
Teaching philosophy and its influence on learning outcomes.

According to Figure 3, educators using student-centered methodologies, like offering feedback and resolving student issues, enhance engagement, motivation, and academic achievement, and also reduce worry and tension. Prioritizing active learning and student accountability promotes increased engagement and cultivates a dynamic, supportive educational atmosphere, which is essential for mental health and well-being.

#### 3.2.3 Professional development and resource challenges

Educators emphasized the need for continuous professional development to stay current with innovative teaching techniques. However, significant challenges arose due to limited resources, such as technology, limited space, and essential materials, all of which hindered effective education. One participant indicates, ‘our equipment is outdated, and we lack the requisite materials for thorough physical education’. While recognizing the need for development, several educators noted that there were limited opportunities for advancement. These findings emphasize the need for improved resources and professional development, thereby mitigating stress and anxiety among educators, ultimately fostering a supportive learning environment for students.

**Figure 4:**
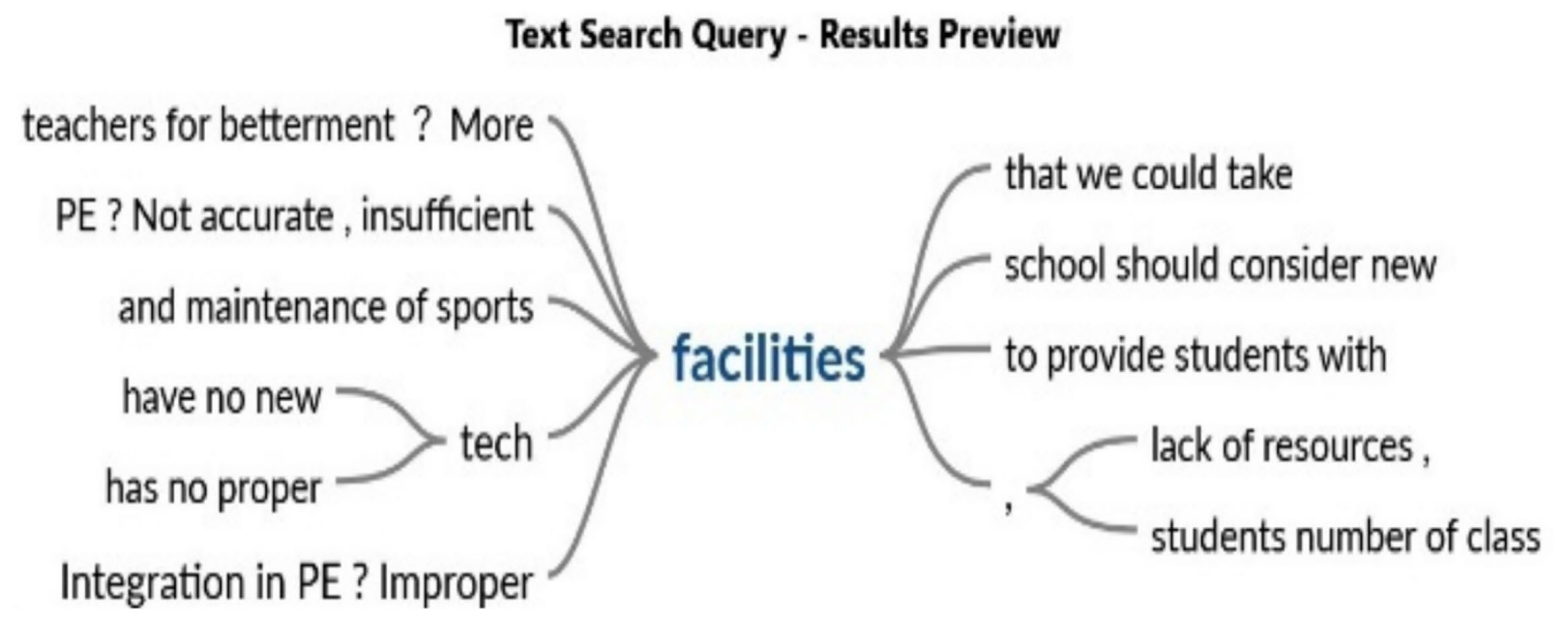
Inadequate facilities.

Figure 4 illustrates that insufficient facilities and obsolete technical resources substantially hinder the effectiveness of physical education training. Educators observed that inadequate equipment, insufficient space, and the absence of essential resources hinder excellent instruction. These are exacerbated by increasing class sizes, hindering the provision of personalized attention and true participation. This disengagement may lead to mental health concerns, including stress, anxiety, and diminished motivation in students and educators, thus impacting the learning environment.

**Figure 5:**
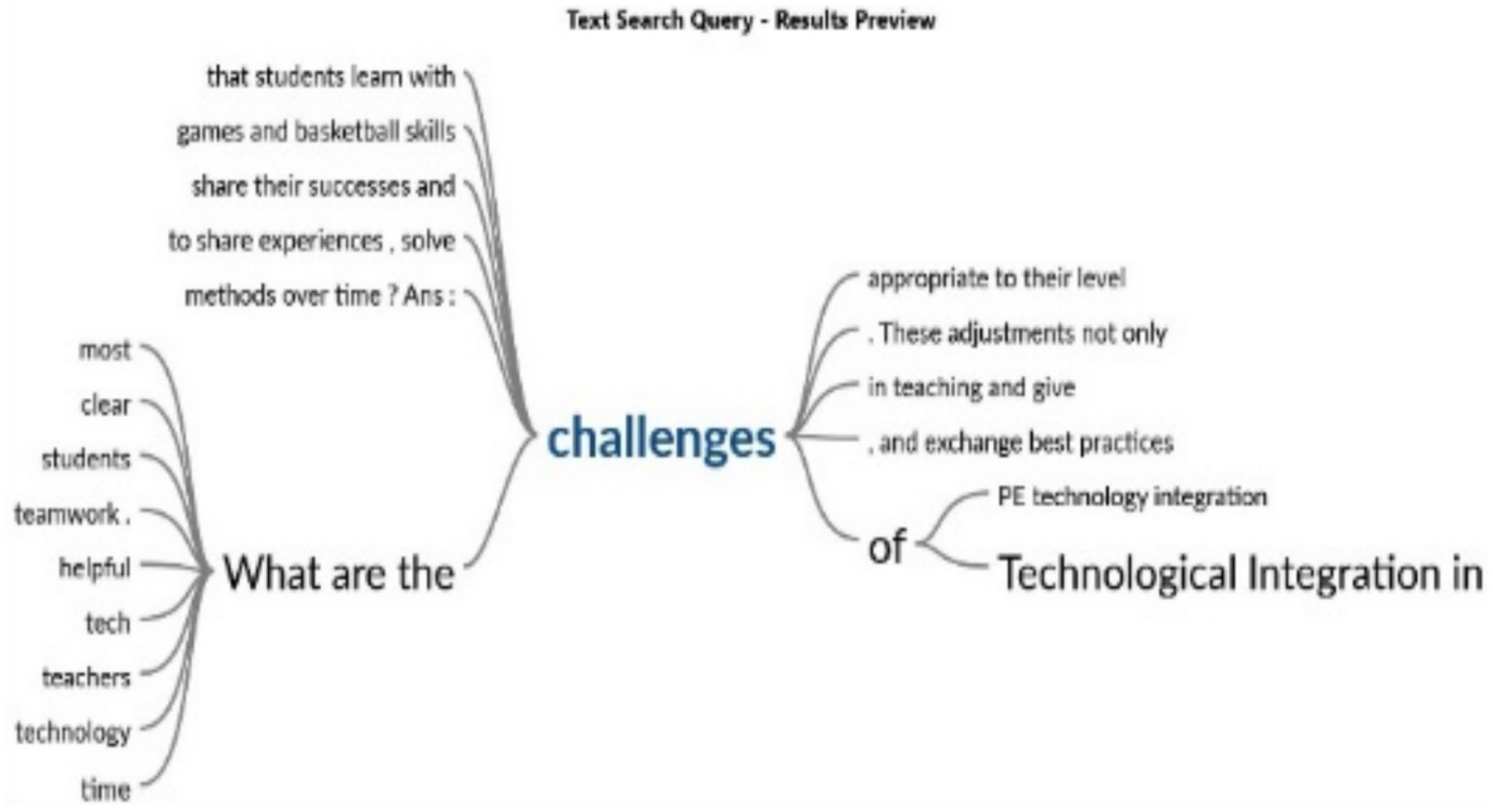
Challenges in incorporating effective learning.

Figure 5 illustrates the obstacles educators face by integrating technology into PE classrooms. These constraints include inadequate technical resources, difficulty in adjusting instructional approaches among student levels, and the professional development of using technology effectively. The effort to overcome these constraints may create tension and anxiety among instructors and students, affecting the educational experience. Addressing these issues by effectively incorporating technology tools into the physical education curriculum is crucial for enhancing engagement, reducing psychological stress, and improving instructional effectiveness.

#### 3.2.4 Student diversity and workforce management

The issue of student diversity highlights the need for educators to adapt their instructional methods to accommodate diverse learning styles and proficiency levels. Educators highlighted that students’ diverse learning and interests involve various educational techniques to promote inclusion. A participant said, ‘I often have to modify activities for students with diverse abilities, which can be difficult in large classes.” Additionally, task management was seen as a crucial element influencing the quality of education. Increasing class sizes and inadequate resources hindered educators’ capacity to provide personalized attention, exacerbating stress and burnout among teachers. An instructor noted that managing large groups with limited resources is challenging, which affects the quality of training. These findings emphasize the importance of effectively managing student diversity and workload to enhance educational outcomes and mitigate mental health stress for students and instructors.

#### 3.2.5 Visual representation of themes

This research was enhanced by several visual aids, such as idea maps and word clouds created using NVivo. The idea map illustrated the interconnections among instructional practices, teaching philosophy, resource challenges, and student diversity. Resource constraints were directly linked to reduced professional development opportunities and limited use of student-centered techniques, thereby affecting mental health outcomes by inducing stress and dissatisfaction among students and educators.

**Figure 6:**
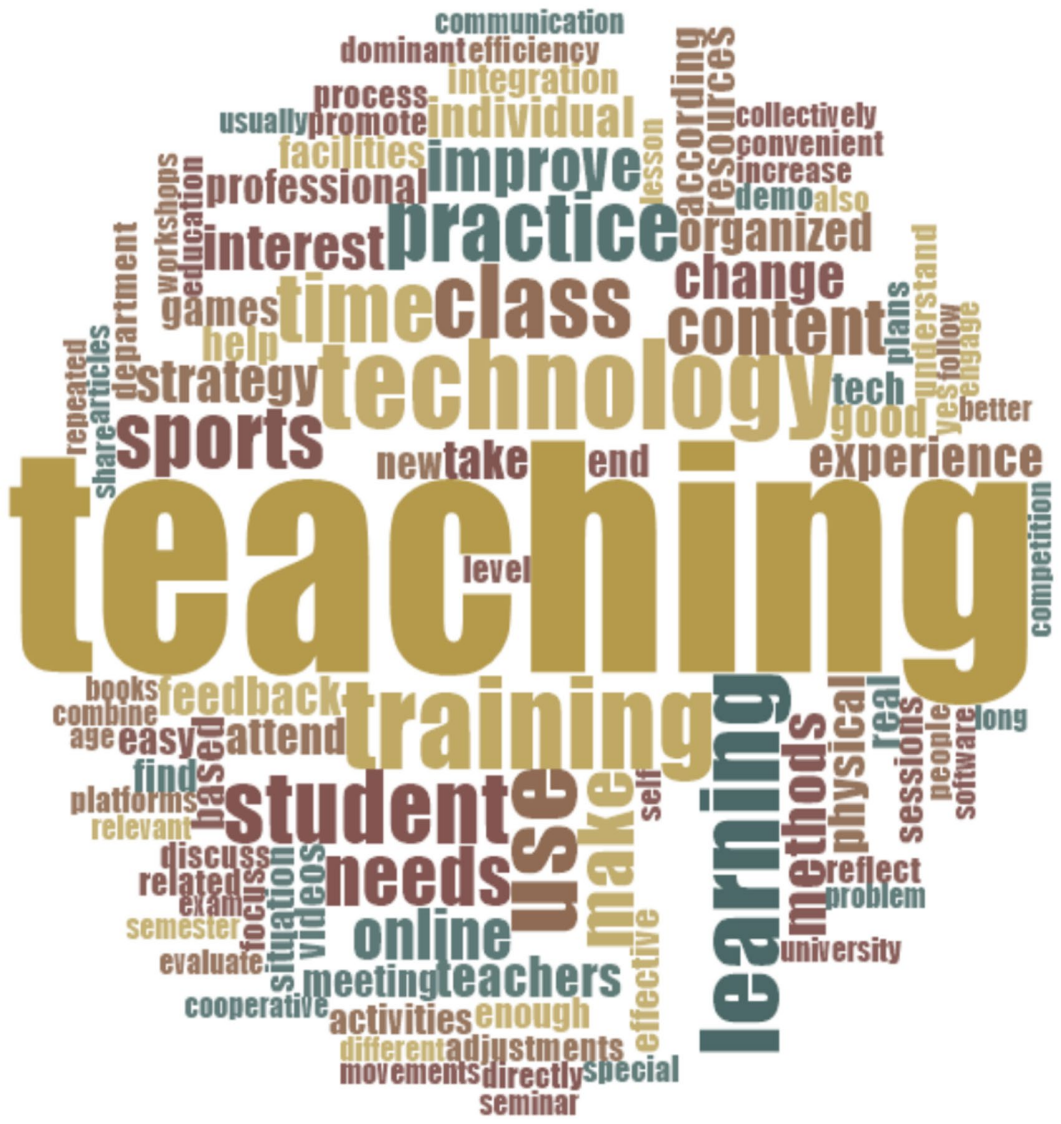
Word cloud of the most frequent terms.

Figure 6 presents a word cloud which emphasizes the most frequently referenced phrases in the interview data. Terms such as ‘instruction, learners, involvement, and evaluation’ were prevalent, highlighting the primary focus of the discussions. These terms highlight the importance of effective teaching practices in enhancing academic success and mental well-being by fostering a positive learning environment.

## 4 Discussion

This study underscores the critical role of physical education (PE) in enhancing both physical and mental health outcomes for university students, particularly in the context of non-athlete students. Physical inactivity is a significant risk factor for cardiometabolic diseases such as cardiovascular diseases and diabetes, which are prevalent in sedentary populations (Singh et al., 2019). Disengagement from PE programs, common among non-athlete students, exacerbates these risks, leading to long-term health consequences. The student-centered teaching methods examined in this study, including cooperative learning and peer teaching, not only improve physical competence but also contribute to better mental health outcomes, reducing symptoms of anxiety and depression.

Furthermore, this study emphasizes the importance of exercise in promoting academic resilience. Previous research has shown that engaging in regular physical activity enhances the ability to cope with academic stress and improves overall mental well-being (Van Ryzin and Roseth, 2021; Casey and Goodyear, 2015). Our findings support this body of literature, demonstrating that student-centered PE interventions can not only prevent chronic diseases but also improve psychological resilience, which is essential for academic success, resulting in improvements in students’ skills and decreased anxiety and depression, echoing that involvement in learning can reduce stress and emotional concerns while promoting engagement (Van Ryzin and Roseth, 2021; Vasconcellos et al., 2020). In contrast, instructor-centered methods had minimal impact on these outcomes, indicating that directive teaching may fail to address students’ motivational and emotional needs (Eppley and Dudley-Marling, 2019; Mason and Otero, 2021). This emphasizes the importance of teaching style, which supports the growing consensus in pedagogy that interactive and autonomy-supportive teaching enhances performance and well-being (Freeman et al., 2014; Munafo, 2016; Haerens et al., 2015).

This study evaluated the effectiveness of pedagogical approaches in PE and examined the impact of professional development, teaching philosophy, and resource constraints. Research indicates that pedagogical practices significantly affect educational outcomes. Despite inconsistencies in data collection rates across institutions, these findings are only related to universities in Henan.

### 4.1 Specialized problem analysis: key findings on teaching strategies

This research indicated that PE instructors at universities in Henan primarily employed CL, direct teaching, and student-centered methodologies. These tactics were uniform across faculty levels, professors, associate professors, and lecturers, corroborating prior study (Casey and Goodyear, 2015). Teaching styles differed by faculty position; professors preferred structured methods, such as demonstrations and personalized training, while associate professors and lecturers preferred more interactive, student-centered approaches. This generational difference in teaching style is noteworthy, which corresponds with studies indicating that senior faculty often use directive tactics owing to their knowledge and classroom management abilities, while younger faculty concentrate on student involvement (Xiao et al., 2022; Xiao et al., 2022). Importantly, student feedback indicated a strong preference for interactive methods. Data from students indicated that peer teaching and cooperative learning were the most valued tactics. Students appreciated their professors’ expertise and clear explanations, but many reported that limited opportunities for interaction and active participation hindered their overall learning experience. When instructors incorporated peer-led activities and teamwork, students responded with higher engagement and motivation, feeling more empowered in the learning process. This shift toward student-centered learning, encouraging autonomy and peer support, is consistent with constructivist educational theories and has been shown to improve outcomes in PE and other fields (Casey and Goodyear, 2015; Munafo, 2016). Our findings reinforce that active learning strategies not only build skills but also cultivate a more positive attitude toward physical activity among non-athlete students (Freeman et al., 2014).

### 4.2 Students’ perspectives on teaching strategies and resource constraints in PE instruction

Beyond teaching style, our discussion considers the contextual challenges that influenced these outcomes. Educators in this study reported significant resource limitations, outdated equipment, inadequate facilities, and large class sizes, which hindered the implementation of high-quality, student-centered instruction. These constraints made it difficult to provide personalized feedback or adapt activities to individual needs, often forcing instructors to revert to basic lecture and demonstration formats. Consistent with additional studies, under-resourced and oversized classes impede effective PE teaching (Lipponen et al., 2022; Kajanus, 2016). Our participants described how such limitations increased their stress and reduced instructional quality. Many instructors found that heavy workloads and institutional pressures educators with large teaching loads and managing many students, were unlikely to employ diverse, interactive techniques. Burdened educators often reverted to more traditional, teacher-centered methods under time constraints. This trend highlights that workload and resource strain can undermine educators’ efforts to engage students, supported by studies linking teacher burnout to diminished use of student-focused practices (Lipponen et al., 2022). Addressing these institutional issues is crucial, as even the most effective pedagogical innovations require a supportive environment to flourish.

### 4.3 Addressing student diversity in PE

Another key finding was the impact of student attitudes and diversity on teaching effectiveness. We observed that non-athlete students, particularly those from certain academic majors (e.g., liberal arts), often viewed physical education as a low-priority, ‘mandatory’ course rather than a growth opportunity. Many of these students were hesitant to fully engage in physical activities, citing discomfort, lack of interest, or the perception that PE was not relevant to their personal or career goals. This reluctance manifested in lower participation and motivation levels, which in turn reduced the effectiveness of certain instructional techniques. For example, one highly structured approach in our study (a detailed skill-breakdown exercise) proved counterproductive for disengaged learners; non-athlete students with low initial confidence became even less motivated when faced with drills that emphasized fundamentals in isolation. Educators had to adjust on the fly, shifting away from such breakdown drills toward more inclusive, enjoyable activities. They reported better success using interactive methods (like cooperative games and peer-led drills) to engage reluctant students and ease their emotional detachment. This observation is in line with self-determination theory and related research: students who perceive an activity as controlling or irrelevant are prone to disengage, whereas approaches that satisfy needs for autonomy, competence, and relatedness can improve motivation (Somerset and Hoare, 2018). Our findings underscore that teachers must adapt to diverse student needs and initial attitudes. Strategies that work for enthusiastic or athletically inclined students may falter with non-athletes who lack fundamental skills or interest. Thus, flexibility in teaching, introducing cooperative team tasks, offering choice in activities, and providing positive reinforcement were essential to achieve our positive outcomes. By creating a more inclusive and supportive atmosphere, instructors helped students overcome negative preconceptions about PE, gradually fostering greater participation and even enjoyment in some cases.

### 4.4 Teacher workload and strategy adjustments

These results indicate that pedagogy and context both influence student outcomes in university PE programs. Student-centered, interactive teaching methods can substantially improve physical skills and mental health indicators for non-athlete students; however, success depends on adequate support. The alignment of these results with prior research adds credibility to these conclusions. Active engagement strategies yielded multifaceted benefits, compared to passive or one-size-fits-all teaching (Casey, 2016; Bailey et al., 2009). These challenges identified resource constraints and student apathy, highlighting that innovative pedagogies are linked to supportive institutional conditions and adaptive approaches to student diversity.

### 4.5 Practical implications for PE curriculum development

Based on our findings, several practical implications emerge for physical education curriculum design in higher education. Firstly, university PE curricula should be updated to intentionally incorporate student-centered and collaborative learning components. The clear benefits of cooperative learning and peer instruction observed in this study suggest that curriculum planners should allocate ample time for group activities, team sports, and peer-led projects, rather than focusing solely on instructor-led drills or competitive athletics. For example, modules can be designed to emphasize team building, problem-solving tasks, and inclusive fitness activities that engage all skill levels. Such an approach can help transform PE classes from obligatory routines into opportunities for personal growth and social connection, which is especially important for non-athlete students. This implication aligns with the concept of lifelong physical education as a curricular goal (Shi, 2023), where students are taught sport techniques and skills to maintain physical and mental well-being throughout their lives. Additionally, mental health and emotional well-being should be explicitly integrated into the PE curriculum objectives. Simple interventions such as brief mindfulness exercises during cooldowns, discussions on stress relief through exercise, or reflective journaling about physical activity experiences can combine the curriculum with mental health outcomes. By making these connections clear, institutions will address the holistic needs of students, reinforcing the idea that physical education can yield cognitive and emotional benefits with physical education. Institutions use evidence-based studies to prioritize engaging, student-centered content that resonates with non-athlete students, thereby improving skill acquisition and attitudes toward physical education.

### 4.6 Professional development for PE instructors

Our study highlights the need for targeted professional development to help physical education instructors adopt modern teaching approaches. The differences observed between senior and junior faculty teaching styles suggest that some educators, particularly those trained under traditional paradigms, would benefit from support in learning and implementing student-centered techniques. Training workshops and continuous education programs should focus on strategies such as CL differentiated instruction and positive youth development methods. Equipping PE teachers with a diverse toolkit of pedagogical approaches will enable them to engage with students more effectively, regardless of interest or skill level. Professional development sessions should include practical demonstrations of how to organize and facilitate group activities and how to integrate discussions of health and well-being into physical skills training. Research has shown that when in-service teachers receive training in cooperative learning models, their effectiveness and willingness to use these methods increase (Jiang et al., 2023). We therefore recommend that universities invest in regular training seminars, peer mentoring, and communities of practice for PE instructors. Senior faculty can be paired with innovative junior colleagues to share practices, creating an environment of mutual learning. Moreover, professional development should extend beyond teaching, focusing on mental health awareness, motivational psychology, and inclusive education. By understanding the mental and emotional dimensions of student engagement, PE teachers can better support students who are anxious, unmotivated, or have diverse needs. In conclusion, a professional development framework, supported by institutional policy, can empower instructors to continuously refine their teaching. This not only improves immediate classroom outcomes but also contributes to instructors’ effectiveness and job satisfaction, creating a positive feedback loop that benefits students and teachers (Erhorn et al., 2020).

### 4.7 Limitations and policy recommendations for institutions

The limitations of this study must also be acknowledged. First, the research was conducted in Henan Province, limiting the generalizability of the results to other regions or populations. Additionally, the study relied on self-reported data, which may be subject to response biases. The sample may also not fully represent the diversity of the student populations, and potential bias could affect the accuracy of the findings. Future studies should aim to include a broader geographical scope and utilize longitudinal or experimental designs to better understand the causal relationships between teaching strategies, physical health outcomes, and mental health. In terms of practical implications, the findings suggest policy-level recommendations for educational institutions and administrators overseeing university PE programs. To facilitate the effective implementation of student-centered teaching, institutions must address structural barriers, including class size, resource allocation, and incentive structures. A key recommendation is to reduce PE class sizes and student-teacher ratios wherever feasible. Smaller classes allow instructors to give more individualized attention, manage learning activities safely and effectively, and build stronger connections with students. These are factors that amplify modern teaching methods. Universities should also invest in upgrading physical education facilities and equipment, as outdated or insufficient resources directly impede teaching quality, as concluded from this study. Providing proper sports equipment, maintaining facilities, and even leveraging technology could enhance learning experience and outcomes. Such investments signal institutional commitment to the value of PE.

Additionally, universities should implement policies that encourage and reward innovative teaching in PE. Allocating dedicated time and funding for PE instructors’ professional development is another crucial policy step. This should involve budget for workshops, providing teaching grants for curriculum innovation, or hiring specialist coaches to train faculty educators in new pedagogical collaboration to integrate mental health initiatives with physical activity programs. Universities should launch wellness campaigns that link PE with mental health resources, thereby reinforcing that physical education is central to student well-being. On a broader scale, education policymakers should consider guidelines or frameworks that embed mental health objectives into national or regional PE curriculum standards, ensuring a holistic approach is demonstrated and adopted. By removing institutional obstacles and enacting supportive policies, administrators can substantially improve the conditions for PE instructors to apply modern teaching approaches, ultimately leading to better student engagement, health, and skill development.

### 4.8 Future research directions

This study contributes to our understanding of how student-centered physical education interventions can enhance physical skill development and mental health outcomes. However, there are several avenues for future research that would deepen our insights and broaden the applicability of these findings.

Future research should employ longitudinal or experimental designs to evaluate the long-term effects of student-centered teaching methods on both physical health and mental well-being. Longitudinal studies, in particular, would allow for tracking the sustained impact of PE interventions on student outcomes, such as improvements in physical fitness, mental health, and cardiometabolic risk over time. Additionally, experimental studies could better establish causal relationships between teaching strategies and mental health outcomes, providing stronger evidence of the effectiveness of PE interventions.

As noted in a recent study, structured exercise and physical therapy have therapeutic benefits for individuals experiencing depression and are effective in reducing suicide risk (Kanani et al., 2025). Beyond immediate improvements in skill acquisition and classroom engagement, previous research has emphasized the mental health benefits of exercise. Future research should incorporate this broader body of literature, exploring how collaborative PE interventions can challenge, such as stress, anxiety, and depression. By integrating mental health outcomes with physical performance measures, future studies could demonstrate the preventive role of PE in managing mental health issues, especially in non-athlete populations.

Extending this research to other regions or countries would provide valuable insights into the effectiveness of student-centered PE interventions across diverse cultural contexts. Cross-cultural studies could assess whether the positive outcomes observed in Henan Province universities are generalizable to other geographical areas or whether cultural differences in attitudes toward PE influence the effectiveness of these teaching strategies. Such studies should allow for a broader understanding of how cultural factors, such as attitudes toward physical activity and mental health perceptions, impact the success of PE programs.

## 5 Conclusion

This study provides evidence that student-centered teaching methods, particularly cooperative learning and peer-led approaches, significantly enhance non-athlete physical skill development and engagement in university PE classes. These interactive strategies were associated with improved academic performance and better mental health outcomes, specifically, reductions in anxiety and depression, whereas traditional direct instruction yielded minimal benefits. Our findings aligned with studies indicating that active, participatory pedagogy produces multifaceted benefits, where passive, one-size-fits-all teaching often falls short. The focus on physical and psychological outcomes offers evidence that modern student-centered PE approaches can serve as a dual intervention, improving both physical fitness and mental well-being in traditionally under-engaged populations.

From a broader educational perspective, these results emphasize the need to modernize university PE programs and ensure that student-centered approaches are accompanied by supportive institutional conditions. Institutions should provide enabling factors such as smaller class sizes, adequate resources, and administrative support for interactive learning to flourish. Investing in instructor training and professional development, especially to assist traditionally trained faculty educators in adopting more collaborative and inclusive practices, is essential. By embedding cooperative learning and mental health awareness into the PE curriculum, educators and policymakers can transform physical education classes into inclusive experiences that foster physical competence, psychological resilience, and ultimately lifelong healthy habits.

## Data Availability

The original contributions presented in the study are included in the article/Supplementary material, further inquiries can be directed to the corresponding author.
