# Supplementary material for: Addressing physical skills and mental health: the role of modern teaching approaches in non-athlete university PE programs
Source: Front Psychol. 2025 Dec 5;16:1664027. doi: 10.3389/fpsyg.2025.1664027 (PMC12715939; doi:10.3389/fpsyg.2025.1664027)
Supplement: Supplementary file 1 [file Supplementary_file_1.docx]

**INTERVIEW**

**Foundational Knowledge**

1. **How would you define a "teaching strategy"? show me some examples, if student have to pass exam in 2 weeks how will he learn strategies quickly)**

**请举例说明，例如学生考试前两周还没有掌握考试内容，您会给他提供什么样的方法帮助其学的很快。**

**Ans:**

1. **If student do not like sports what method you use to make them like sports?**

**如果学生不喜欢体育，曾使用什么方法让其喜欢？**

**Ans:**

1. **How do you stay updated on the latest teaching techniques and strategies in physical education?**

**您如何及时了解最新的体育教学技术和策略?**

**Ans:**

1. **Can you describe a teaching strategy you recently learned and implemented?**

**您能描述一下您最近学习和实施的教学策略吗？**

**Ans:**

1. **Which teaching strategies do you believe are most essential for a PE teacher, and why?**

**您认为哪些教学策略对于体育教师来说是最重要的，为什么？**

**Ans:**

**Practical Application**

1. **Describe a time when you had to modify a teaching strategy based on the needs or responses of your students.**

**描述一次您必须根据课堂上学生的需要或反应调整教学策略的经历.**

**Ans:**

1. **How do you measure the effectiveness of a particular teaching strategy in your classes?**

**您如何衡量课堂上特定教学策略的有效性？**

**Ans:**

1. **Can you provide an example of a strategy that was particularly successful? Conversely, one that didn’t yield the expected results?**

**您能否提供一个特别成功的策略示例？或者相反地，一个没有达到预期的结果的策略示例？**

**Ans:**

1. **How do you handle situations where students might not respond well to a certain teaching strategy?**

**如果学生对某种教学策略反应不佳，您如何处理这种情况？**

**Ans:**

**Personal and Professional Development**

1. **How often do you attend workshops or training sessions related to teaching strategies?**

**您多久参加一次与教学策略相关的研讨会或培训课程？**

**Ans:**

1. **Can you share an example of a particularly beneficial workshop?**

**您能分享一个特别有益的研讨会的例子吗？**

**Ans.**

1. **Does the PE department organize meetings for teachers for self-analyze?**

**体育系是否组织教师进行自我剖析会议？**

**Ans:**

1. **How do you engage with other PE teachers for mutual professional growth?**

**您如何与其他体育老师合作以实现共同的专业成长？**

**Ans:**

1. **How long did you learn new strategies write down the time?**

**写下你学习新策略的时间有多久?**

**Ans:**

1. **Are there any specific resources or organizations that you rely on for continuous learning in PE?**

**您是否依赖任何特定资源或组织来继续学习体育?**

**Ans:**

1. **How do you leverage technology for your professional development in PE?**

**您如何利用技术来促进体育教育的专业发展？**

**Ans:**

1. **Have you ever been mentored in teaching techniques, or have you mentored others? Can you describe that experience?**

**您是否曾经接受过教学技巧方面的指导，或者您指导过其他人吗？你能描述一下那次经历吗？**

**Ans:**

1. **How do you deal with feedback from students regarding your teaching methods??**

**您如何处理学生对您的教学方法的反馈?**

**Ans:**

**Teaching Philosophy and Strategy Integration**

1. **How would you describe your teaching philosophy, and how does it influence your choice of teaching strategies?**

**您如何描述您的教学理念？它是如何影响您对教学策略的选择的?**

**Ans:**

1. **Could you share your approach for incorporating feedback, self-reflection, and assessment into enhancing your teaching methods over time?**

**您能否分享一下您如何将反馈、自我反思和评估融入到不断改进的教学方法中？**

**Ans:**

**Specific Scenarios**

1. **Describe how you would approach a class with a mix of skilled athletes and complete beginners. What strategies would you employ?**

**描述一下您将如何开展由熟练运动员和完全初学者混合组成的课程。你会采用什么策略？**

**Ans:**

1. **Describe a challenging teaching scenario you encountered and the strategies you used to address it.**

**描述您遇到的具有挑战性的教学情景以及您用于解决该情景的策略?**

**Ans:**

**Future Oriented**

**Whether you will change your strategies in future, what will lead you change your strategies?**

**未来您是否会改变策略，什么会导致您改变策略？**

**Ans:**

1. **How do you plan to evolve or improve your understanding and implementation of teaching strategies in the future?**

**您计划如何发展或改进您在未来对教学策略的理解和实施？**

**Ans:**

1. **In what ways do you intend to enhance your teaching methods moving forward?**

**今后您打算通过哪些方式改进您的教学方法?**

1. **What are your short-term and long-term professional development goals as a PE teacher?**

**作为体育老师，您的短期和长期职业发展目标是什么?**

1. **Any other information you want to give, or the suggestion for PE department and teachers for betterment？**

**您还有什么想提供的信息，或者对体育部门和老师的改进建议?**

**Credibility:**

**1.What are the characteristics of a credible PE teacher?**

**值得信赖的体育老师有哪些特征？**

**Ans:**

**2.what personal qualities make PE teacher credible in the eyes of student?**

**什么个人品质使体育老师在学生眼中可信？**

**Ans:**

**3.How do students characterize PE teacher credibility?**

**学生如何评价体育老师的可信度？**

**Ans:**

**Technological Integration:**

1. **Determine the role of technology in teaching strategies.**

**确定技术在教学策略中的作用。**

**Ans:**

1. **what are the benefits of Technological Integration in PE?**

**什么是** **PE技术整合的好处？**

**Ans:**

1. **What are the challenges of Technological Integration in PE?**

**PE技术整合面临哪些挑战？**

**Ans:**
