# Supplementary material for: Addressing physical skills and mental health: the role of modern teaching approaches in non-athlete university PE programs
Source: Front Psychol. 2025 Dec 5;16:1664027. doi: 10.3389/fpsyg.2025.1664027 (PMC12715939; doi:10.3389/fpsyg.2025.1664027)
Supplement: Supplementary file 2 [file Supplementary_file_2.docx]

**Questionnaire 3**

**BASED ON LEARNING**

**基于学习**

**1. Demographic Information**

**1.人口统计信息**

**Age: ____**

**年龄：____**

**2. Gender: Male / Female / Prefer not to say / Other: ________**

**2. 性别：男/女/不愿说/其他：________**

**3. Number of years/months being coached: ________**

**3. 接受辅导的年/月数：________**

**4. which sports do you play in PE class?**

**你在体育课上进行哪些运动？**

**Ans:**

**4. Rate the clarity of the PE teaching strategies on a scale of 1 to 5 (1 being very unclear, 5 being very clear).**

**3.用1到5的量表对体育教学策略的清晰度进行评分（1分非常不清楚，5分非常清楚）。**

**___ 1**

**___ 2**

**___ 3**

**___ 4**

**___ 5**

1. **How often do you understand the purpose behind a specific drill or exercise?**
2. **Always**
3. **Often**
4. **Sometimes**
5. **Rarely**
6. **Never**

**4. 你多久了解一次特定训练或练习的目的？**

**a.始终**

**b.经常**

**c.有时**

**d.很少**

**e.从不**

**5. Do you feel the PE teaching strategies employed are effective in improving your skills/performance?**

1. **Strongly Agree**
2. **Agree**
3. **Neutral**
4. **Disagree**
5. **Strongly Disagree**

**5.你觉得所采用的体育教学策略对提高你的技能/表现有效吗？**

**a.强烈同意**

**b.同意**

**c.中性**

**d.不同意**

**e.强烈反对**

**6. Do you believe the teacher communicates the strategies clearly?**

1. **Always**
2. **Often**
3. **Sometimes**
4. **Rarely**
5. **Never**

**6.你认为老师能清楚地传达策略吗？**

**a.始终**

**b.经常**

**c.有时**

**d.很少**

**e.从不**

**7. When you're unsure about a strategy or drill, how comfortable do you feel asking the coach for clarification?**

1. **Very Comfortable**
2. **Comfortable**
3. **Neutral**
4. **Uncomfortable**
5. **Very Uncomfortable**

**7.当你对策略或训练不确定时，你会觉得向教练寻求解释有多舒适？**

**a.非常舒适**

**b.舒适**

**c.中立**

**d.不舒适**

**e.非常不舒适**

**8. How often are you provided with feedback about your performance?**

1. **After every session**
2. **Weekly**
3. **Monthly**
4. **Rarely**
5. **Never**

**8.你多久收到一次关于你表现的反馈？**

**a.每次会议后**

**b.每周**

**c.每月**

**d.很少**

**e.从不**

**9. Do you think the feedback provided helps you understand the PE teaching strategies better?**

1. **Strongly Agree**
2. **Agree**
3. **Neutral**
4. **Disagree**
5. **Strongly Disagree**

**9.你认为所提供的反馈有助于你更好地理解体育教学策略吗？**

**a.强烈同意**

**b.同意**

**c.中立**

**d.不同意**

**e.强烈反对**

**10. Which coaching strategy or method has been most beneficial for you?**

**[Open-ended answer]**

**10.哪种指导策略或方法对你最有利？**

**[开放式回答]**

**11. Are there any PE teaching strategies that you find confusing or less effective? If so, please describe.**

**[Open-ended answer]**

**11.有没有什么体育教学策略让你感到困惑或效果不佳？如果是，请描述。**

**[开放式回答]**

**12. What would you suggest to improve the communication or clarity of PE teaching strategies?**

**[Open-ended answer]**

**12.你有什么建议可以提高体育教学策略的交流或清晰度？**

**[开放式回答]**

**13. Any additional comments or feedback about the teaching strategies?**

**[Open-ended answer]**

**13.你对教学策略有任何其他意见或反馈吗？**

**[开放式回答]**

**New questions**

**14. Did the teacher provide enough feedback during the main activity?**

- No feedback (0)
- Limited feedback (1)
- Some helpful feedback (2)
- Clear and helpful feedback (3)

**15. In your opinion, which teaching strategies are most effective in helping you learn new skills in PE class? (Choose all that apply)**

- Teacher-centred: Teacher explains and demonstrates skills, then students practice.
- Student-centred: Students work collaboratively or independently to explore and practice skills.
- Technology-integrated: Technology tools (apps, interactive games) are used to enhance learning.
- Differentiated instruction: Instruction is tailored to individual needs and learning styles.
- Other (please specify): ___________________

**16. In your opinion, which teaching strategies make PE classes more interesting and enjoyable? (Choose all that apply)**

- Games and competitions
- Creative activities and challenges
- Variety of activities and equipment
- Opportunities for choice and self-expression
- Positive and encouraging feedback from teachers
- Other (please specify): ___________________

1. **17. For each statement below, please rate the frequency of occurrence in your PE classes on a scale of 1 (Never) 2 (often) 3 (sometimes) 4 (Rarely) to 5 (Always):** Statement | Frequency (1-5) ---|---|

- Teachers explain instructions clearly before activities. |
- Teachers break down skills into smaller steps for easier learning. |
- Teachers provide opportunities for students to practice skills in different ways. |
- Teachers offer different activities to cater to different skill levels. |
- Teachers give individual feedback on my performance. |
- Teachers use technology tools to enhance learning in PE classes. |
- We participate in discussions or group activities related to health and fitness. |

**18. Are there any specific learning styles or preferences you have that are not currently being addressed by your PE teachers' teaching strategies? If so, how could they be better accommodated?**

**Ans:**

**19. Do you feel like all students in your PE class have equal opportunities to learn and participate effectively? Why or why not?**

**Ans:**

**20. What was the most challenging part of PE lesson, and how could it be improved?**

**21.**  **Do you feel you had enough opportunities to ask questions during the lesson? (Circle one) Yes / No / Unsure**

**22. Did the activities challenge you appropriately? (Circle one) Too Easy / Just Right / Too Difficult**
